# Supplementary material for: Protocol for a randomized clinical trial comparing the efficacy of Structured Diet (SD) and Regular Therapy (RT) for adolescents with malnutrition having Autism Spectrum Disorder (ASD)
Source: PLoS One. 2023 Nov 29;18(11):e0292326. doi: 10.1371/journal.pone.0292326 (PMC10686458; doi:10.1371/journal.pone.0292326)
Supplement: S1 File — (PDF) [file pone.0292326.s001.pdf]

Sample Size Calculator

Determines the minimum number of subjects for adequate study power

Study Group Design

vs.
Two independent study groups

vs.
One study group vs. population

Two study groups will each receive different treatments.

Primary Endpoint

Dichotomous (yes/no)

Continuous (means)

The primary endpoint is an **average**.
Eg, blood pressure reduction (mmHg), weight loss (kg)

Statistical Parameters

Anticipated Means

Group 1 ? 4.7 ± 1.7

Group 2 ? 25 %

% Decrease

Enrollment ratio ? 1

Type I/II Error Rate

Alpha ? 0.05

Power ? 80%

Reset Calculate

RESULTS

Continuous Endpoint, Two Independent Sample Study

Sample Size	
Group 1	33
Group 2	33
**Total**	**66**

Study Parameters

Mean, group 1	4.7
Mean, group 2	3.53*25% dec*
Alpha	0.05
Beta	0.2
Power	0.8

View Power Calculations

$$k = \frac{n_2}{n_1} = 1$$

$$n_1 = \frac{(\sigma_1^2 + \sigma_2^2/K)(z_{1-\alpha/2} + z_{1-\beta})^2}{\Delta^2}$$

$$n_1 = \frac{(1.7^2 + 1.7^2/1)(1.96 + 0.84)^2}{1.17^2}$$

$$n_1 = 33$$

$$n_2 = K * n_1 = 33$$

$\Delta = |\mu_2 - \mu_1|$ = absolute difference between two means
σ_1, σ_2 = variance of mean #1 and #2
n_1 = sample size for group #1
n_2 = sample size for group #2
α = probability of type I error (usually 0.05)
β = probability of type II error (usually 0.2)
z = critical Z value for a given α or β
k = ratio of sample size for group #2 to group #1

About This Calculator

This calculator uses a number of different equations to determine the minimum number of subjects that need to be enrolled in a study in order to have sufficient statistical power to detect a treatment effect.[1]

Before a study is conducted, investigators need to determine how many subjects should be included. By enrolling too few subjects, a study may not have enough statistical power to detect a difference (type II error). Enrolling too many patients can be unnecessarily costly or time-consuming.

Generally speaking, statistical power is determined by the following variables:

- **Baseline Incidence:** If an outcome occurs infrequently, many more patients are needed in order to

detect a difference.

- **Population Variance:** The higher the variance (standard deviation), the more patients are needed to demonstrate a difference.
- **Treatment Effect Size:** If the difference between two treatments is small, more patients will be required to detect a difference.
- **Alpha:** The probability of a type-I error -- finding a difference when a difference does not exist. Most medical literature uses an alpha cut-off of 5% (0.05) -- indicating a 5% chance that a significant difference is actually due to chance and is not a true difference.
- **Beta:** The probability of a type-II error -- not detecting a difference when one actually exists. Beta is directly related to study power (Power = 1 - β). Most medical literature uses a beta cut-off of 20% (0.2) -- indicating a 20% chance that a significant difference is missed.

Post-Hoc Power Analysis

To calculate the post-hoc statistical power of an existing trial, please visit the **post-hoc power analysis calculator (Power.aspx)**.

References and Additional Reading

1. Rosner B. *Fundamentals of Biostatistics*. 7th ed. Boston, MA: Brooks/Cole; 2011.

Search

Enter search term ...

Related Calculators

- Post-hoc Power Calculator (Power.aspx)

Follow Us!

(http://www.facebook.com/clincalc) (http://twitter.com/clincalc) (http://feeds.feedburner.com/ClinCalc)

Get Email Updates (http://eepurl.com/mXpuL)

New and Popular

- Opioid Equianalgesic Calculator (/Opioids)
- ClinCalc DrugStats 2021 Update – The Most Commonly Prescribed Drugs in the United States (//clincalc.com/blog/2021/09/clincalc-drugstats-2021-update-the-most-commonly-prescribed-drugs-in-the-united-states/)
- Acute Physiology and Chronic Health Evaluation (APACHE II) Calculator (/IcuMortality/APACHEII.aspx)
- RxHero for iOS: Educational Gaming of the Top 250 Drugs (//clincalc.com/blog/2019/04/rxhero-for-ios-educational-gaming-of-the-top-250-drugs/)
- ASCVD Risk Calculator (/Cardiology/ASCVD/PooledCohort.aspx)
- RapidASCVD: Calculate ASCVD scores seriously fast (//clincalc.com/blog/2020/08/rapidascvd-calculate-ascvd-scores-seriously-fast/)
- DrugStats Database (/DrugStats)
- Vancomycin Calculator (/Vancomycin)

Open Menu

Cite This Page

Show AMA citation

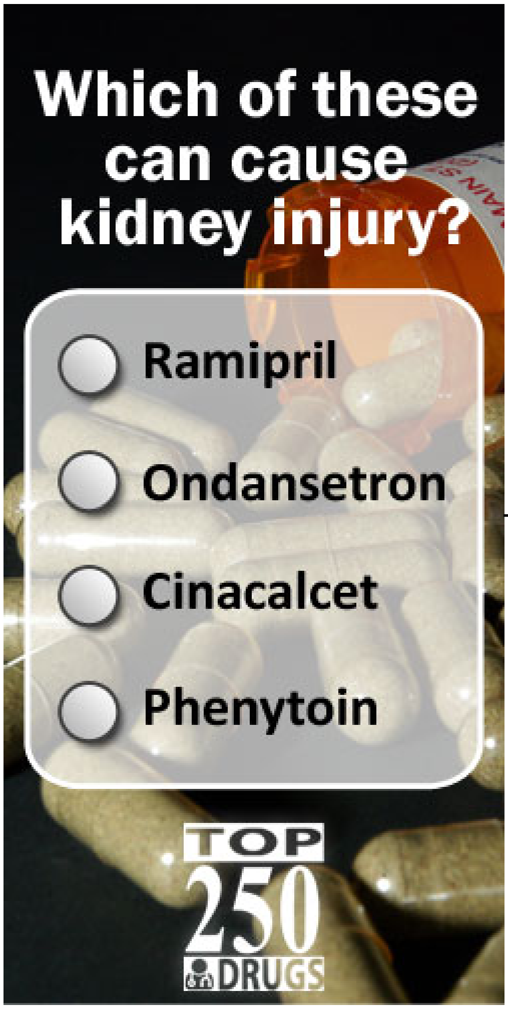

(//clincalc.com/academy)

©2023 - ClinCalc LLC. All rights reserved.

Updated Jul 24, 2019
