# Supplementary material for: Protocol for a randomized clinical trial comparing the efficacy of Structured Diet (SD) and Regular Therapy (RT) for adolescents with malnutrition having Autism Spectrum Disorder (ASD)
Source: PLoS One. 2023 Nov 29;18(11):e0292326. doi: 10.1371/journal.pone.0292326 (PMC10686458; doi:10.1371/journal.pone.0292326)
Supplement: S2 File — (PDF) [file pone.0292326.s002.pdf]

Annexure 1

Study Design: Cross sectional survey on the Children of Autism in Bangladesh

Population: Children with ASD in Bangladesh.

Sample size: According to UNICEF, there are 64000000 children aged bellow 18 years in Bangladesh and the literature assumes 7.5 every 10000 children in Bangladesh. With this prevalence there are nearly 48000 children with autism in Bangladesh. According to EPI Info Version 7.2.0 from the CDC of the USA, a total of 384 participants from all the divisions with 48 participants per division can provide 95% confidence interval with 1.0 design effect at 5% acceptable margin of error and 50% reach of expected frequency of random sampling to the targeted population.

Sampling Technique: Stratified Random Sampling

- Every division is a stratum
- From every strata we will random the serial number of the centers and schools to randomly assign the data collection center
- From the random list we will prioritize the centers according to computer based random allocation priority.
- We will continue data collection until the targeted 48 participants are reached
- We may recruit more than the targeted participants to improve external generalization

Sampling Frame

Dhaka Division:		
SN	Setup	Contact
1	Centre for the Rehabilitation of the Paralysed (CRP)<br>Chapin, Savar, Dhaka-1343	Letter, ERC
2	Autistic Children's Welfare Foundation (ACWF)<br>Address: House 74, Lane 3, Block E, Mirpur 12, Dhaka	Letter<br>01914403331
3	Beautiful Mind<br>Address: Plot 1145, Road 6/A, Dolipara, Sector 5, Uttara, Dhaka	Letter<br>02-8917699
4	IPNA School	Letter

	Institute of Pediatric Neurodisorder & Autism<br>Bangabandhu Sheikh Mujib Medical University, Dhaka	
5	School for Gifted Children<br>Address: Tauri Foundation, House 6/9, Block E, Lalmatia, Mohammadpur, Dhaka	Letter<br>01713173667
6	Society for the Education & Inclusion of the Disabled (SEID)<br>Address: Humayun Road, Mohammadpur, Dhaka	Letter<br>01730059555
7	Society for the Welfare of Autistic Children (SWAC)<br>Address: 70/Ka, Pisciculture, Shyamoli, Dhaka	Letter<br>02-8118836
8	Society for the Welfare of the Intellectually Disabled (SWID)<br>Address: 4/A Eskaton Garden, Dhaka	Letter<br>02-8319438
9	প্রয়াস (বিশেষায়িত শিক্ষা প্রতিষ্ঠান)<br>ঢাকা সেনানিবাস, ঢাকা-১২০৬	০১৭৬৯-০১৭-৭২২<br>৮৭১৫২৩০
10	সুইড বাংলাদেশ (প্রধান কার্যালয়)<br>৪/এ ইস্কাটন গায়ডনে, ঢাকা-১০০০	৯৩৩৪০০৯<br>৯৩৫৬৫৯২<br>৯৩৫০০২৬
11	কেন্দ্রীয় প্রতিষ্ঠান (এনআইআইডি)<br>এ/২, সুবর্ণ ভবন, মিরপুর-১৪, ঢাকা-১২০৬	০১৭২৮-২২৮-৭৮৮<br>০১৮১৯০৯৯০৯১
12	সুইড ল্যাবরেটরী মডেল স্কুল<br>৪/এ ইস্কাটন গার্ডেন, ঢাকা-১০০০	০১৭১৬-৫৪৬-৫৭০
13	রমনা বুদ্ধি প্রতিবন্ধী বিদ্যালয়<br>রমনা শাখা, ৪ ইস্কাটন গার্ডেন, ঢাকা-১০০০	০১৭১৬-১৯২-০৮৯
14	মিরপুর বুদ্ধি প্রতিবন্ধী বিদ্যালয়<br>১০/১০, ২য় তলা, মিরপুর-১১ ১/২, পল্লবী, ঢাকা।	০১৭৫৪-২৮৮-১৮৮<br>০১৯৫০-৪৭৪-২১৩
15	ধানমন্ডি বুদ্ধি প্রতিবন্ধী বিদ্যালয়,<br>ধানমন্ডি শাখা ৮/১০ ব্লক-এ, লালমাটিয়া, ঢাকা।	০১৯২০-৭৮৩-১৪৭
16	তেজগাঁও বুদ্ধি প্রতিবন্ধী বিদ্যালয়<br>২৫৫ পশ্চিম আগারগাঁও , শেরে বাংলা নগর, ঢাকা।	০১৭৫২-০৭৬-৫৫০
17	বুদ্ধি প্রতিবন্ধী বিদ্যালয় ,	০১৬৭৩-৯৪৯-০৯০

	ধামরাই শাখা, পুরনো সি, ভবন, যাত্রাবাড়ী , ধামরাই, ঢাকা	
18	খিলগাঁও বুদ্ধি প্রতিবন্ধী বিদ্যালয়<br>(খিলগাঁও সরকারি উচ্চ বিদ্যালয় সংলগ্ন) খিলগাঁও -তালতলা, ঢাকা-১২১৯	০১৭৩২-৩৪১-৪৭৬
19	বুদ্ধি প্রতিবন্ধী বিদ্যালয়<br>৩৫/২/ডি দ্বীন নাথ সেন রোড, ঘুন্টিঘর, গেন্ডারিয়া, ঢাকা।	০১৭২৪-০৯৫-১৩১<br>০১৯৩২-৪৪৬-১৭০
20	কল্যাণী ইনক্লুরসভ স্কুল<br>৬ বড়বাগ, সেকশন-২, মিরপুর, ঢাকা-১২১৬	০১৯৯২-৮৪৩-৮৫৫<br>ফেরদৌসি মৌওলা<br>০১৭১১-৯৩৪-৭৬১
21	কল্যাণী ইনক্লুরসভ স্কুল<br>১২ নিউ সার্কুলার রোড, পশ্চিম মালিবাগ, ঢাকা-১২০৭	০১৯১৪-৭৫১-৮৫৯
22	কল্যাণী ইনক্লুরসভ স্কুল<br>বালিথা শাহবেলীশ্বর, ধামরাই, ঢাকা।	০১৯৩৩-৯০৬-৮৩৪<br>০১৭১২-২৫১-৪২০
23	বাংলাদেশ প্রতিবন্ধী ফাউন্ডেশন<br>কল্যাণী ইনক্লুরসভ স্কুল<br>নলাম (বাগবাড়ি), মির্জানগর, আশুলিয়া, সাভার,ঢাকা	০১৯১৫-৬২৭-৪১৯
24	কল্যাণী ইনক্লুরসভ স্কুল<br>চরনগরদী, পলাশ, নররসাংদী	০১৭১৮-১৬১-০৫৯
25	কল্যাণী ইনক্লুরসভ স্কুল<br>মারিয়া, স্বল্পমারিয়া, কিশোরগঞ্জ সদর, কিশোরগঞ্জ	০১৯২১-৭০৮-২৬৭<br>০১৭১৮-৬১২-৩০৯
26	কল্যাণী ইনক্লুরসভ স্কুল<br>বাগাট, মধুখালী, ফরিদপুর।	০১৯১৮-২১২-১৪২<br>০১৭১২-৯৪৬-৬২৫
27	বুদ্ধি প্রতিবন্ধী বিদ্যালয়<br>তারাত মেলা সড়ক, কুঠিবাড়ী, কমলাপুর, ফররদপুর।	০১৭৪০-৫৫৫-০৩৩
28	নারায়নগঞ্জ শিক্ষক,<br>শাহনেওয়াজ, চেম্বর-১৪৩, বি. বি রোড, পলি ক্লিনিক, নারায়নগঞ্জ।	০১৯৩৭-৯৩০-১৮৪
29	টাঙ্গাইল বুদ্ধি প্রতিবন্ধী বিদ্যালয়<br>টাঙ্গাইল শাখা স্টেডিয়াম পাড়া, টাঙ্গাইল	০১৭৩৬-৭৮১-৬১১<br>০১৭৩৬-০১৩-৭৬৮
30	বুদ্ধি প্রতিবন্ধী ও অটিরস্টক বিদ্যালয়<br>মালাউড়ি, মধুপুর, টাঙ্গাইল	০১৭২৫-০৬৪-৯০৯
31	বুদ্ধি প্রতিবন্ধী বিদ্যালয়<br>পুবাইল শাখা, বড় কয়ের, পুবাইল, গাজীপুর	০৭২৬-৪০৭-৮৫৮
32	প্রশিসেস প্রতিবন্ধী বিদ্যালয়	০১৯৫১-৩৭৯-৮৫৯

	শহীদ বাচ্চু সড়ক, মাদারীপুর সদর মাদারীপুর	
33	বর্ণ প্রতিবন্ধী বিদ্যালয়<br>৩২৯ পাওয়ার হাউজ রোড, গোপালগঞ্জ	০১৭২৭-৭৪১-৩৯১
34	JPUF মিরপুর	০১৭১২৬৬৬৬০২
35	JPUF লালবাগ	০১৭১৮১০৪৩০৭
36	JPUF উত্তরা	০১৭১৮০৬৯২৩৩
37	JPUF যত্রাবাড়ী	০১৭১৪৬৫৯০৭১
38	JPUF নারায়ণগঞ্ঞ	০১৮১৩২০৮১৭৯
39	JPUF শরিয়তপুর	০১৯২০৯৫৯৯৫৬
40	JPUF ধামরাই	০১৭১৯৬৪৩৮৪১
41	JPUF কাপাশিয়া	০১৭৫৪১২০১৪৮
42	JPUFমুন্সিগঞ্জ	০১৭০৫০৫৭৩৭০
43	JPUF টুঙ্গিপাড়া	০১৭১৭০৮৩৫১৩
44	JPUF টাঙ্গাইল	০১৭২১৪৪৫১৪৪
45	JPUF ফরিদপুর	০১৭৩৮৯৭৩৯২২
46	JPUF নরসিংদী	০১৭১২০৩৫৩০৩
47	JPUF কিশোরগঞ্জ	০১৭১৯৩২২৫১৫
48	JPUF রাজবাড়ী	০১৫৫৩৬৬৩৬৯২
49	JPUF মধুপুর	০১৭৪৩৬৩৩৩৭৮
50	JPUF ভাংগা	০১৬৭৩৩৬৯৫৪৫
51	JPUF রাজৈর	০১৭১৬৩৯০৭১৭
52	JPUF মানিকগঞ্জ	০১৭১৬২৯৯২৯২
53	JPUF গাজীপুর	০১৭৮০৩৫৪৪২২
54	JPUF ভৈরব	০১৭১০০৭৮৪৩৩
55	JPUF সিংগাইর	০১৭৪৬১৯৬৮৮০
56	JPUF গোপালগঞ্জ	০১৭৪০৯৬৫২৬৫
57	JPUF মাদারীপুর	০১৭৮৪৪০০৩৪১

Chittagong Division:

SN	Setup	Contact
58	Autistic Children's Welfare Foundation (ACWF)<br>Address: Mirzapool, Muradpur, Chittagong	Letter<br>01819324579
59	Matrisneha Autism Children Academy<br>Address: 128 Panchlaish Residential Area, Chittagong	Letter<br>01712-011653
60	Seher Autism Center<br>Address: Hill View Housing Society, House 17, Road 2, East Nasirabad, Chittagong	Letter<br>01918704200
61	বুদ্ধি প্রতিবন্ধী বিদ্যালয়<br>চট্টগ্রাম শাখা মিয়া আমান উল্ল্যাহ তবন, জাকির হসাইন রড, চট্টগ্রাম - ৪২০২	০১৭১৮-০০৪-৯১৫
62	বুদ্ধি প্রতিবন্ধী বিদ্যালয়<br>চাঁদপুর শাখা, সালাম মঞ্জিল, বিপুনিবাগ, চাঁদপুর	০১৭২১-২৫৬-১০৮<br>০১৭৫৪-২৮৫-৮৯০
63	বুদ্ধি প্রতিবন্ধী স্কুল, ফেনী শাখা মিজান রোড, ফেনী	০১৯৯৯-০৪২-১২৭
64	আছমাতুন্নেছা বুদ্ধি প্রতিবন্ধী ও অটিস্টিক বিদ্যালয়<br>গ্রামঃ কালাই শ্রীপাড়া, জেলাঃ ব্রাহ্মণবাড়িয়া	০১৭২৭-২৭৯-৭৭৯
65	সনি বুদ্ধি প্রতিবন্ধী বিদ্যালয়<br>বিজেশ্বর, উলাচাপাড়া, ব্রাহ্মণবাড়িয়া	০১৭১২-৩৬৬-০২৮
66	বুদ্ধি প্রতিবন্ধী ও অটিস্টিক বিদ্যালয়<br>নোয়াখালী শাখা, নোয়াখালী জেলা শিল্পকলা একাডেমী	০১৭১৪-৬৫৩-৫৭৩
67	নাঙ্গলকোট বুদ্ধি প্রতিবন্ধী বিদ্যালয়<br>নাঙ্গলকোট শাখা, নাঙ্গলকোট পৌরসভা, কুমিল্লা	০১৯১৩-৯৮০-১০৮
68	কুমিল্লা বুদ্ধি প্রতিবন্ধী ও অটিস্টিক বিদ্যালয়	০১৭৫১-৫৫৫-৭০০
69	JPUF চট্টগ্রাম	০১৮৪৮৩২৪৫৬৯
70	JPUF বরুডা	০১৯১৪৬১৩৪৬২
71	JPUF রাঙ্গুনিয়া	০১৭১৮৬১৯৩৯৬
72	JPUF খাগড়াছড়ি	০১৭১৫৯৭৭৪৩৮
73	JPUF ব্রাহ্মণবাড়িয়া	০১৭০৯৬৪৩৮৬০
74	JPUF আখাউড়া	০১৭১২২৯৮০৪০
75	JPUF চাঁদপুর	০১৯১৬৫৮০২৩৪

76	JPUF ফেনী	০১৮১৩৫৮৮৮১১
77	JPUF লক্ষ্মীপুর	০১৭১৬২০৮০০৩
78	JPUF রাঙ্গামাটি	০১৫৫৩২৪৯৯০৭
79	JPUF কুমিল্লা	০১৭৫৯৯৫৭৩০৫
80	JPUF ফরিদগঞ্জ	০১৬৮৮৬২৩৪১০
81	JPUF মনোহরগঞ্জ	০১৮১৮৪৪৮৫৪৩
82	JPUF হাতিয়া	০১৮১২৩৪৫৬৬৭
83	JPUF কক্সবাজার	০১৮১৮৫৩০৮১০
84	JPUF বান্দরবান	০১৯১২৯০২২০০
85	JPUF নোয়াখালী	০১৬৭৬২০৩৩৫৯
86	JPUF টেকনাফ	০১৮২৭২০১৫২৬

Rajshahi Division

SN	Setup	Contact
87	School for Gifted Children<br>Address: 3367, Seroil , Rajshahi	Letter<br>01710060322
88	রাজশাহী বুদ্ধিপ্রতিবন্ধি ও অটিস্টিক বিদ্যালয়<br>বাজে কাজলা, পোস্ট কাজলা, থানা মতিহার, জেলা রাজশাহী	০১৯২৬-৪৩৮-০৯৩
89	বগুড়া বুদ্ধি প্রতিবন্ধী ও অটিস্টিক বিদ্যালয়<br>ডালিয়া হাউস, মালতিনগর, মাটির মসজিদ লেন, বগুড়া	০১৭১৫-০৪১-৭৫৯
90	পাবনা বুদ্ধি প্রতিবন্ধী বিদ্যালয়<br>আতাইকুলা রোড, শালগাড়িয়া, পাবনা	০১৭২৭-৩৯৪-০১০
91	JPUF রাজশাহী	০১৭১৯৪৭৩০৪৫
92	JPUF সিরাজগঞ্জ	০১৭২১৬০৫৪২৪
93	JPUF চাঁপাইনবাবগঞ্জ	০১৭১৬৩৮৫৮৮৮
94	JPUF বাঘা	০১৭২৯১৫১৬২৮
95	JPUF বগুড়া	০১৭৩৪৭৫৫১১১
96	JPUF নাটোর	০১৭১৯৪৭৪১২
97	JPUF জয়পুরহাট	০১৭২৬২২৪৬৮৭
98	JPUF নওগাঁ	০১৫৫৬৩০০৬৩৪

99	JPUF আত্রাই	০১৭২৭৬৬১৫১০
100	JPUF পাবনা	০১৭২৪৪০৪৮০৪

Rangpur Division

SN	Setup	Contact
101	বুদ্ধি প্রতিবন্ধী বিদ্যালয়<br>রংপুর শাখা, লায়ন্স স্কুল এন্ড কলেজ, জি এল রায় রোড, রংপুর	০১৭৪৪-৩২৬-৭৯৪
102	বুদ্ধি প্রতিবন্ধী বিদ্যালয় পায়রাবন্দ, মিঠাপুকুর, রংপুর	০১৭১৮-৮০৫-৭৪৬
103	গোলেজা খাতুন বুদ্ধি প্রতিবন্ধী ও অটিস্টিক বিদ্যালয়<br>গোবিনাথপুর শাখা, পীরগঞ্জ, রংপুর	০১৭১৩-৭৩৭-১২৮
104	লালমনিরহাট বুদ্ধি প্রতিবন্ধী বিদ্যালয়<br>লালমনিরহাট শাখা, স্টেশন রোড, লালমনিরহাট	০১৭১৬-৯৬৫-৮৫৯
105	বুদ্ধি প্রতিবন্ধী বিদ্যালয়<br>আদিতমারি শাখা,<br>সাং-নায়েকটারী, পোঃ সাপ্টিবাড়ী, থানাঃ আদিতমারী, জেলাঃ লালমনিরহাট	০১৭৪৫-২৩০-৭৮৬
106	নর্থ বেঙ্গল বাক ও শ্রবণ প্রতিবন্ধী বিদ্যালয়<br>গোশালা, সোসাইটি চত্বর, সদর, লালমনিরহাট	০১৭৬১-৮৫৫-৩০৭
107	দিনাজপুর প্রতিবন্ধী ও অটিস্টিক বিদ্যালয়<br>দিনাজপুর শাখা, ক্ষেত্রীপাড়া, দিনাজপুর	০১৭১৬-১৫৩-৪২৫<br>০৫৩১-৫২৮২১
108	বুদ্ধি প্রতিবন্ধী বিদ্যালয় নাগেশ্বরী শাখা, মহিলা কলেজ পাড়া, কুড়িগ্রাম	০১৭১৩-৭৩১-৭২৭
109	বুদ্ধি প্রতিবন্ধী বিদ্যালয় সরকারি শিশু পরিবার সংলগ্ন, কৃষ্ণপুর, হাসপাতালপাড়া, কুড়িগ্রাম	০১৭৫০-২৮৫-৭৬৫
110	রিতা আক্তার বানু (লুৎফা) বুদ্ধি প্রতিবন্ধী বিদ্যালয়<br>রমনা, উপজেলায়-চিলমারী, কুড়িগ্রাম	০১৭১০-৪০০-২২০
111	গাইবান্ধা বুদ্ধি প্রতিবন্ধী বিদ্যালয়<br>সুন্দরজাহান মোড়, গাইবান্ধা	০১৭১৪-৭৫৫-০৯৪
112	ভারতখালি অটিজম ও বুদ্ধি প্রতিবন্ধী বিদ্যালয়<br>ডাকঘরঃ ভারতখালি, উপজেলাঃ সাঘাটা, জেলাঃগাইবান্ধা	০১৭১৮-৪৭৭-৬১০
113	আরিফ আফজালুর রাব্বি বুদ্ধি প্রতিবন্ধী বিদ্যালয়<br>গ্রামঃ উদাখালী, পোস্টঃ ফুলবাড়ী, জেলাঃ গাইবান্ধা	০১৯১৮-১৬৬-৭৭৩

114	কালিকাপুর বুদ্ধি প্রতিবন্ধী বিদ্যালয়<br>ডাকঘরঃ কালিকাপুর, উপজেলাঃকিশোরগঞ্জ,<br>জেলাঃ নীলফামারী	০১৭৩৭-৮৯৩-২৮৮<br>০১৭৬৮-৯৪০-৫০৮
115	ফ্রীড এন্ড ফ্রীড মাতৃছায়া অটিস্টিক শিশু নিকেতন<br>আশ্রমপাড়া, আর্টগ্যালারি, ঠাকুরগাঁও	০১৭১৬-৯২০-৪৩৯
116	JPUF রংপুর	০১৭১৭২৪৭৪৬৪
117	JPUF দিনাজপুর	০১৭১৭৭২২৬৯২
118	JPUF গাইবান্ধা	০১৯১৪১৭১০৬১
119	JPUF লালমনিরহাট	০১৭৩৬২৯৫৮৫৬
120	JPUF ভুরুঙ্গামারী	০১৭২৩০৯০৯০০
121	JPUF পীরগঞ্জ	০১৭৩৭৯১৮৩৭৯
122	JPUF নীলফামারী	০১৯২১৫৬৫৫৫০
123	JPUF ঘোড়াঘাট	০১৭২৩০৭৪৩৯৭
124	JPUF কুড়িগ্রাম	০১৯১৪৯৮৪৮৩০
125	JPUF ঠাকুরগাঁও	০১৭৩৯১৯৫৩৩২
126	JPUF পঞ্চগড়	০১৭১১৩৯০৯২৮
127	JPUF ফুলবাড়ী	০১৭১৭৩৩৯১৩৯
128	JPUF হরিপুর	০১৯২৩৯৭৩৮০০
129	JPUF দেবিগঞ্জ	০১৭১৫৪১২০৪৭

Mymensingh Division

SN	Setup	Contact
130	বুদ্ধি প্রতিবন্ধী ও অটিস্টিক বিদ্যালয়<br>ময়মনসিংহ শাখা, ৭ শ্যামাচরণ রায় রোড, ময়মনসিংহ	০১৭১২-৩৬৪-৫০৮
131	প্রভাতী প্রতিবন্ধী বিদ্যালয় চেচুয়া, উপজেলা মুক্তাগাছা, জেলা-ময়মনসিংহ	০১৭২৫-৮৫৩-৭৬৩
132	সরিষাবাড়ী বুদ্ধি প্রতিবন্ধী ও অটিস্টিক বিদ্যালয় সরিষাবাড়ী শাখা, বীর ধানাটা, সরিষাবাড়ী, জামালপুর	০১৭১৮-২০২-৬০৩
133	বুদ্ধি প্রতিবন্ধী ও অটিস্টিক বিদ্যালয়<br>জামালপুর শাখা, (সিং হাজানি কাচারীপাড়া মডেল সরকারি প্রাথমিক বিদ্যালয় সংলগ্ন), জামালপুর	০১৭৩৫-২৫৭-৮৭৬
134	কামরুন্নেছা আশরাফ বুদ্ধি প্রতিবন্ধী ও অটিস্টিক বিদ্যালয়<br>মুক্তিযোদ্ধা মেহের আলী সড়ক (চন্দ্রনাথ উচ্চ বিদ্যালয়), সদর, নেত্রকোনা	০১৭১৭-৩৩৮-৫৯৯

135	JPUF ময়মনসিংহ	০১৯২১৩০৩০৫৭
136	JPUF ভালুকা	০১৭১৯১৮২৬৮৩
137	JPUF নেত্রকোণা	০১৭১৮৭২৫০৮৬
138	JPUF হালুয়াঘাট	০১৭৫৩৮৮১৯৫৪
139	JPUF জামালপুর	০১৭২০৫১৭৭৮৮
140	JPUF শেরপুর	০১৭১৬৪০৩৮৯০
141	JPUF মোহনগঞ্জ	০১৭১৭৫৫১৫৮৯

Khulna Division

SN	Setup	Contact
142	বুদ্ধি প্রতিবন্ধী বিদ্যালয়<br>কুষ্টিয়া শাখা, ৩০/১,বরদা রায় রোড, ১৯ নং ওয়ার্ড, খুলনা	০১৭১৫-৩১২-৭৮২<br>০১৭১২-৯৮৪-১০৮
143	বুদ্ধি প্রতিবন্ধী বিদ্যালয়<br>কুষ্টিয়া শাখা, বিচারপতি মাহবুব মোর্শেদ সড়ক, জেলখানার মোড়, কুষ্টিয়া	০১৭১৮-৫০৪-৫৬৯
144	মহল্লা প্রতিবন্ধী প্রাথমিক বিদ্যালয়<br>শিমুলিয়া, খোকসা, কুষ্টিয়া	০১৭৬১-৭১৯-২৮৬
145	বুদ্ধি প্রতিবন্ধী ও অটিস্টিক বিদ্যালয়<br>সাতক্ষীরা শাখা, কলেজ রোড, রাজার বাগান, সাতক্ষীরা	০১৭৪০-৮৫৮-০৪৬
146	বুদ্ধি প্রতিবন্ধী বিদ্যালয় উপজেলা মোড, আশাশুনি সাতক্ষীরা	০১৭২১-১৯৭-৪১৫
147	সুইট বুদ্ধি প্রতিবন্ধী বিদ্যালয় বাগেরহাট শাখা, জেলখানা রোড, বাগেরহাট-৯৩০০	০১৯১১-১০৬-২০২
148	আলহাজ্ব ডাঃ মোঃ মোজাম্মেল হোসেন বুদ্ধি প্রতিবন্ধী বিদ্যালয়, মোড়েলগঞ্জ, বাগেরহাট	০১৭১৯-৫০৫-১২৭
149	বুদ্ধি প্রতিবন্ধী বিদ্যালয়<br>ঘোপ নাওয়াপাড়া রোড, (সারাদিন অফিসের পাশে), যশোর	০১৭১৬-৪৩৪-৪৬৭
150	JPUF খুলনা	০১৭১৭৫২৮৩৩৪
151	JPUF যশোর	০১৮১৪৯০২৬৬৮
152	JPUF নড়াইল	০১৭২১১৮৩৩৬৪
153	JPUF মোরেলগঞ্জ	০১৭৭৭৯৫১৪০৪
154	JPUF মেহেরপুর	০১৭০৯৬৪৩৮৫৯
155	JPUF বাগেরহাট	০১৯১৩৯২০০৩৭
156	JPUF রুপসা	০১৯১৬৬১১৮২৫

157	JPUF মাগুরা	০১৭১২৫২৩১৮৯
158	JPUF সাতক্ষীরা	০১৯১৮২৯২৬৯৩
159	JPUF চুয়াডাংগা	০১৭৪৭৭৭৪১৯১
160	JPUF কুষ্টিয়া	০১৭৩৭৬০৫৭৭৭
161	JPUF ঝিনাইদহ	০১৯২১৮৩১৬৭৭
162	JPUF শৈলকুপা	০১৯১৭৪৯৬৩৬৯

Barisal Division

SN	Setup	Contact
163	বুদ্ধি প্রতিবন্ধী বিদ্যালয়,<br>মল্লিক রোড, বরিশাল	০১৭১২-০৮৭-৪৩৪
164	বুদ্ধি প্রতিবন্ধী ও অটিস্টিক বিদ্যালয়<br>পিরোজপুর শাখা,জেলা স্কাউট ভবন, পিরোজপুর	০১৯৯২-৯৮৪-৪২৬
165	সুইড বাংলাদেশ বুদ্ধি প্রতিবন্ধী বিদ্যালয়<br>ভান্ডারিয়া, পিরোজপুর	০১৭২০-৩৫৩-২৮৬
166	পটুয়াখালী বুদ্ধি প্রতিবন্ধী বিদ্যালয়<br>সরকারি কলেজ রোড, (সমাজসেবা অফিস সংলগ্ন), পটুয়াখালী	০১৭১৯-০৫৮-৬৫৪
167	বাংলা স্কুল, সদর রোড, ভোলা	০১৭১১-৯৫২-৪১৯<br>০১৬২৯-৭৯৩-৪০৯
168	কানুদাসকাঠী প্রতিবন্ধী বিদ্যালয় গালুয়া বাজার, থানাঃরাজাপুর,<br>জেলাঃঝালকাঠি	০১৭১১-৩৮৩-১৫২
169	JPUF মাঠবারিয়া	০১৭২১৬০৬৩২৪
170	JPUF পিরোজপুর	০১৭৩২৭৯১১২০
171	JPUF ভোলা	০১৭১৬৩০৪৩৯২
172	JPUF ঝালকাঠী	০১৭১২৮১২৮৯৮
173	JPUF বরগুনা	০১৭১৯৮১৭০৬৯
174	JPUF বরিশাল	০১৭১৯০৮৮৯৫৮
175	JPUF পটুয়াখালী	০১৭১২২৩৬৬৯০
176	JPUF নলছিটি	০১৭১৬৮০৯২৬৯
177	JPUF পাথরঘাটা	০১৮৩৫৮১১৮০৫

Sylhet Division

SN	Setup	Contact
178	বুদ্ধি প্রতিবন্ধী বিদ্যালয়<br>সিলেট শাখা, শেখঘাট, সিলেট	০১৭৩৯-৯৯৬-৯৬৭
179	সিলেট আর্ট এন্ড অটিস্টিক স্কুল, কুমারপাড়া, সিলেট	০১৭১২-৭৩৭-৩৯৯
180	সিলেট ইনক্লুসিভ স্কুল,বাগবাড়ী, সিলেট	০১৭৭২-২২৭-৫৯৫
181	ব্লুমিং রোজেস বুদ্ধি প্রতিবন্ধী ও অটিস্টিক স্কুল<br>মৌলভীবাজার শাখা, উপজেলার সংলগ্ন, সদর মৌলভীবাজার	০১৭১২-৪৫৯-৮৬৮
182	JPUF সিলেট	০১৭২২০২৭৯৪৯
183	JPUF মৌলভীবাজার	০১৭৩৪৩৩৫৬৫৫
184	JPUF চুনারুঘাট	০১৭৩০৯৩৫৪১৪
185	JPUF হবিগঞ্জ	০১৭৪৬৫১০৮৬৯
186	JPUF মাধবপুর	০১৭২৩৪৮৪০৯০
187	JPUF রাজনগর	০১৯১৩৫৫২২৬০
188	JPUF বিশ্বনাথ	০১৯১১৮১৮০৪৯
189	JPUF সুনামগঞ্জ	০১৯১১১৯৬৭৭৩

Computer generated random sequence

Dhaka	
14	0.01457
49	0.014745
45	0.025736
4	0.035049
37	0.066128
8	0.069582
41	0.087452
55	0.095276
25	0.095372
18	0.099835
2	0.128794
57	0.141644
34	0.166339
22	0.171476
5	0.197895
42	0.224599

12	0.250247
35	0.313971
11	0.338912
10	0.349657
20	0.358818
50	0.390067
31	0.437872
46	0.441979
44	0.462862
56	0.485268
48	0.486069
36	0.489108
15	0.515363
53	0.522017
7	0.573997
21	0.587544
1	0.61071
38	0.622078
17	0.633244
24	0.638877
51	0.644468
47	0.659494
13	0.664237
27	0.680447
23	0.699308
9	0.722458
6	0.723068
28	0.758919
29	0.788129
40	0.793499
33	0.820899
26	0.830574
32	0.836935
30	0.855557
52	0.914825
39	0.919384
19	0.924403
16	0.946107
54	0.948465

3	0.962112
43	0.986905

Chittagong	
67	0.007139
74	0.043045
60	0.048
71	0.090834
64	0.197651
63	0.198752
79	0.205887
80	0.208859
68	0.229429
85	0.251534
62	0.278938
70	0.324829
58	0.456195
84	0.481254
66	0.482209
78	0.514012
82	0.524855
81	0.527806
86	0.59404
83	0.660449
61	0.666374
72	0.672867
59	0.705686
73	0.776892
65	0.781552
77	0.864217
76	0.894205
69	0.959067
75	0.983688

Rajshahi	
93	0.097924
88	0.284799
94	0.42084
97	0.435795

92	0.440094
87	0.499289
99	0.572882
91	0.59778
96	0.607243
90	0.684372
89	0.820505
100	0.861134
98	0.908856
95	0.931354

Rangpur	
109	0.020164
128	0.024598
129	0.108474
101	0.109396
121	0.118495
110	0.167233
111	0.183108
107	0.192929
108	0.202108
123	0.213293
127	0.213526
113	0.276125
119	0.370497
115	0.394831
125	0.46473
118	0.468417
106	0.474816
116	0.477297
122	0.483083
105	0.490834
104	0.539866
102	0.562416
114	0.67696
126	0.773105
117	0.792846
120	0.806104
124	0.85021

103	0.854659
112	0.898358

Mymensingh	
132	0.030899
140	0.191585
137	0.395723
136	0.457547
135	0.505616
130	0.634854
134	0.674568
139	0.74819
138	0.748523
131	0.927408
133	0.954821
141	0.995313

Khulna	
157	0.035403
146	0.047306
160	0.051889
148	0.065502
149	0.126981
147	0.13365
151	0.133741
144	0.140314
158	0.259493
153	0.285794
156	0.35139
155	0.389437
150	0.397823
159	0.422203
161	0.472372
142	0.653405
143	0.766046
145	0.777651
154	0.783998
162	0.91817
152	0.97157

Barishal	
176	0.170131
174	0.191823
163	0.203776
165	0.242876
172	0.338739
177	0.362238
166	0.378346
167	0.407498
170	0.455997
173	0.480813
169	0.60971
171	0.6507
175	0.818386
164	0.909318
168	0.973154

Sylhet	
187	0.046448
179	0.088456
185	0.134021
183	0.190164
178	0.338149
186	0.377006
181	0.416546
182	0.421774
184	0.691588
188	0.721403
180	0.733613
189	0.968814
