# Supplementary material for: Protocol for a randomized clinical trial comparing the efficacy of Structured Diet (SD) and Regular Therapy (RT) for adolescents with malnutrition having Autism Spectrum Disorder (ASD)
Source: PLoS One. 2023 Nov 29;18(11):e0292326. doi: 10.1371/journal.pone.0292326 (PMC10686458; doi:10.1371/journal.pone.0292326)
Supplement: S1 Protocol — (PDF) [file pone.0292326.s003.pdf]

Clinical Trial Details (PDF Generation Date :- Wed, 08 Mar 2023 03:55:28 GMT)

**CTRI Number**	CTRI/2022/11/047653 [Registered on: 25/11/2022] - **Trial Registered Prospectively**
**Last Modified On**	24/11/2022
**Post Graduate Thesis**	Yes
**Type of Trial**	Interventional
**Type of Study**	Physiotherapy (Not Including YOGA)<br>Other (Specify) [Nutritional Consultation]
**Study Design**	Randomized, Parallel Group Trial
**Public Title of Study**	Effect of Dilatory advice and exercise therapy for Autism
**Scientific Title of Study**	Effectiveness of Comprehensive Nutritional and Rehabilitation Interventions on improving Stereotypical behavior for Autism Spectrum Disorder

Secondary IDs if Any

Secondary ID	Identifier
NA	NIL

Details of Principal Investigator or overall Trial Coordinator (multi-center study)

Details of Principal Investigator	
**Name**	Ruksana Akter
**Designation**	PhD Student
**Affiliation**	Jashore University of Science & Technology
**Address**	Department of Nutrition and Food Technology Jashore University of Science and Technology, Jashore-7408, Bangladesh.<br><br><br>7408<br>Other
**Phone**	8801711195174
**Fax**	
**Email**	ruksana.bela@gmail.com

Details Contact Person (Scientific Query)

Details Contact Person (Scientific Query)	
**Name**	Md Ashrafuzzaman Zahid
**Designation**	Associate Professor
**Affiliation**	Jashore University of Science & Technology
**Address**	Department of Nutrition and Food Technology Jashore University of Science and Technology, Jashore-7408, Bangladesh.<br><br><br>7408<br>Other
**Phone**	8801718868631
**Fax**	
**Email**	ashraf@just.edu.bd

Details Contact Person (Public Query)

Details Contact Person (Public Query)	
**Name**	Ruksana Akter
**Designation**	PhD Student
**Affiliation**	Jashore University of Science & Technology
**Address**	Department of Nutrition and Food Technology Jashore University of Science and Technology, Jashore-7408, Bangladesh.<br><br><br>7408<br>Other

	**Phone**	8801711195174
	**Fax**	
	**Email**	ruksana.bela@gmail.com

Source of Monetary or Material Support

**Source of Monetary or Material Support**
**>** Mymensingh College of Physiotherapy & Health Sciences, Mymensingh, Bangladesh

Primary Sponsor

**Primary Sponsor Details**	
**Name**	Ruksana Akter
**Address**	Vice-principal, Mymensingh College of Physiotherapy & Health Sciences, Mymensingh, Bangladesh
**Type of Sponsor**	Other [Self]

Details of Secondary Sponsor

**Name**	**Address**
NIL	NA

Countries of Recruitment

**List of Countries**
Bangladesh

Sites of Study

**Name of Principal Investigator**	**Name of Site**	**Site Address**	**Phone/Fax/Email**
Md Imran Hossain	Bela Rehabilitation Solution Point	Navana Tower (Level 21, Flat-B), Gulshan Circle-1, Dhaka 1212., Dhaka, Bangladesh<br>Not Applicable<br>N/A	8801748946284<br><br>imran.bela07@gmail.com

Details of Ethics Committee

**Name of Committee**	**Approval Status**	**Date of Approval**	**Is Independent Ethics Committee?**
BELA Rehabilitation Solution Point	No Objection Certificate	24/11/2022	No
Institute of Physiotherapy, Rehabilitation & Research (IPRR)	Approved	18/10/2022	No

Regulatory Clearance Status from DCGI

**Status**	**Date**
Not Applicable	No Date Specified

Health Condition / Problems Studied

**Health Type**	**Condition**
Patients	Autistic disorder

Intervention / Comparator Agent

**Type**	**Name**	**Details**
Intervention	Rehabilitation and Nutritional Consultation	In treatment group participants will receive Rehabilitation interventions and Nutritional consultation. Rehabilitation interventions include physiotherapy, occupational therapy and speech and language therapy. Intervention will provide by expert PT, OT, SLT and Nutritionist who has experience of working with ASD child. Treatment dosage will depend on child age, ASD symptoms and severity. Physiotherapy includes Balance training- Land based & swimming-based exercises,

			Gait training- Gait training to improves gross motor function and coordination of autism, Coordination practice, Hippotherapy, Gross motor function practice, Proprioception training, Aerobic exercise training, Muscle strengthening. Speech and Language Therapy Treatment: Expert speech and language therapist will provide treatment based of ASD symptoms. Common treatment will include- Applied Behavior Analysis (ABA), Applied behavior Consequence (ABC), Relationship Development Intervention (RDI), Cognitive Behaviour Intervention (CBI), Social communication intervention (e.g Social Story), Picture Exchange, Communication System (PECS), Augmentative and alternative communication (AAC), Visual schedule. Occupational therapy include Sensory integration and 10 areas will encompass under 3 main areas that consist of (i) providing the child with environmental modifications and sensory opportunities during the treatment session, (ii) fostering adaptive responses and providing the just-right challenge, and (iii) promoting the therapist–child relationship, Gross Motor function- exercise will include peg-board activities, dexterity, puzzles, ball play, balancing/vestibular and sensory integration training for children with autism spectrum disorder,Community mobility or travel training, Cognitive behavioral therapy Structured Diet: The nutritionist will provide detailed advice. Diet plan will depend child age, height, BMI. Other comorbid disease and allergy of child to specific foods and physical activity level will consider. The major guiding principles of the dietary plans will include, Healthy, Gluten-Free, Casein-Free, Soy-Free Diet: 1. Adequate intake of a variety of vegetables (including leafy greens) and fruit (preferably whole fruit). 2.

		Adequate protein quality and intake. 3. Adequate, but not excessive, caloric intake. 4. Minimal consumption of “junk” foods and replacement with healthy snacks. 5. Healthy, gluten-free diet, casein-free, and soy-free (HGCSF) e.g. chicken, fish, meat. fruits, vegetables. potatoes, rice, infant rice cereal. 6. Avoidance of artificial flavors, colors, and preservatives 7. Vitamin or Mineral Supplement: Vitamin D supplement by Pediatrician, if needed. Rehabilitation will be provided 3 sessions a day (PT, OT, SLT), 2 days a week for 12 weeks, and Nutritional consultation will be given once in every 2 weeks for 12 weeks.
Comparator Agent	Rehabilitation Interventions	In control group participants will receive Rehabilitation interventions only. Rehabilitation interventions include physiotherapy, occupational therapy and speech and language therapy. Intervention will provide by expert PT, OT, SLT and Nutritionist who has experience of working with ASD child. Treatment dosage will depend on child age, ASD symptoms and severity. Physiotherapy includes Balance training- Land based & swimming-based exercises, Gait training- Gait training to improves gross motor function and coordination of autism, Coordination practice, Hippotherapy, Gross motor function practice, Proprioception training, Aerobic exercise training, Muscle strengthening. Speech and Language Therapy Treatment: Expert speech and language therapist will provide treatment based of ASD symptoms. Common treatment will include- Applied Behavior Analysis (ABA), Applied behavior Consequence (ABC), Relationship Development Intervention (RDI), Cognitive Behaviour Intervention (CBI), Social communication intervention (e.g Social Story), Picture Exchange, Communication System (PECS), Augmentative and

		alternative communication (AAC), Visual schedule. Occupational therapy include Sensory integration and 10 areas will encompass under 3 main areas that consist of (i) providing the child with environmental modifications and sensory opportunities during the treatment session, (ii) fostering adaptive responses and providing the just-right challenge, and (iii) promoting the therapist–child relationship, Gross Motor function- exercise will include peg-board activities, dexterity, puzzles, ball play, balancing/vestibular and sensory integration training for children with autism spectrum disorder,Community mobility or travel training, Cognitive behavioral therapy Rehabilitation will be provided 3 sessions a day (PT, OT, SLT), 2 days a week for 12 weeks.

Inclusion Criteria

Inclusion Criteria	
**Age From**	3.00 Year(s)
**Age To**	22.00 Year(s)
**Gender**	Both
**Details**	1) Diagnosed/Suspected Autism Spectrum Disorder as per ICD 10<br>2) Have nutritional issues according to the screening with Child Nutrition Questionnaire<br>3) Behavioral issues as per the screening Gilliam Autism Rating Scale (GARS-2)

Exclusion Criteria

Exclusion Criteria	
**Details**	1) Parental unacceptance of either Rehabilitation and/or Nutritional Consultation<br>2) incompleted treatment for 12 weeks.

**Method of Generating Random Sequence**	Computer generated randomization
**Method of Concealment**	Sequentially numbered, sealed, opaque envelopes
**Blinding/Masking**	Outcome Assessor Blinded

Primary Outcome

Outcome	Timepoints
Behavioral Status by Gilliam Autism Rating Scale (GARS-2)	12 weeks (3 months) and Follow up after 6 months

Secondary Outcome

Outcome	Timepoints
Nutritional status by Body Mass Index and Anthropometric measures	12 weeks (3 months) and Follow up after 6 months

**Target Sample Size**	**Total Sample Size=**100<br>**Sample Size from India=**0<br>**Final Enrollment numbers achieved (Total)=**Applicable only for Completed/Terminated trials<br>**Final Enrollment numbers achieved (India)=**Applicable only for Completed/Terminated trials
**Phase of Trial**	N/A
**Date of First**	No Date Specified

**Enrollment (India)**	
**Date of First Enrollment (Global)**	04/01/2023
**Estimated Duration of Trial**	**Years=**0<br>**Months=**6<br>**Days=**0
**Recruitment Status of Trial (Global)**	Not Yet Recruiting
**Recruitment Status of Trial (India)**	Not Applicable
**Publication Details**	NA
**Brief Summary**	Through this research, we want to find out the effectiveness of Comprehensive Nutritional and Rehabilitation Interventions on improving Stereotypical behavior for Autism Spectrum Disorder<br><br>**Objectives of the study-**<br><br>To,<br><br>1) Determine the socio-demographics related to malnutrition for ASD in Bangladesh.<br><br>2) Explore the effect of Comprehensive Nutritional and Rehabilitation Interventions behavioral indices of ASD children.<br><br>3) Evaluate the outcome of Comprehensive Nutritional and Rehabilitation Interventions on nutritional status of ASD child in Bangladesh.<br><br>4) To find out the short-term and long-term efficacy of Comprehensive Nutritional and Rehabilitation Interventions for children with ASD.<br><br>**Method:** Prospective Randomized Control Trial (RCT)
