# Supplementary material for: Protocol for a randomized clinical trial comparing the efficacy of Structured Diet (SD) and Regular Therapy (RT) for adolescents with malnutrition having Autism Spectrum Disorder (ASD)
Source: PLoS One. 2023 Nov 29;18(11):e0292326. doi: 10.1371/journal.pone.0292326 (PMC10686458; doi:10.1371/journal.pone.0292326)
Supplement: Attachment — Submitted filename: To Authors for ASD Study.docx [file pone.0292326.s004.docx]

**Protocol for a Randomized Clinical Trial Comparing the Efficacy of Structured Diet (SD) and Regular Therapy (RT) for Children and Adolescents with Malnutrition having Autism Spectrum Disorder (ASD)**

1. Can you clearly stated please, how to evaluate or determine the socio-demographics related to malnutrition for ASD?
2. Can you defined the specific ASD types and their criteria which will be involved in the study?
3. Clearly state the reason why shall you select the sample only from one single center?
4. Did you calculated the sample size for this RCT? If yes then clearly state this.
5. You mentioned that you have received partial grant but why you did not acknowledge this?
6. You mention that you are doing study for Children and Adolescents with Malnutrition having Autism Spectrum Disorder (ASD) but why you selected 13 to 18 years aged participants.
