# Supplementary material for: Protocol for a randomized clinical trial comparing the efficacy of Structured Diet (SD) and Regular Therapy (RT) for adolescents with malnutrition having Autism Spectrum Disorder (ASD)
Source: PLoS One. 2023 Nov 29;18(11):e0292326. doi: 10.1371/journal.pone.0292326 (PMC10686458; doi:10.1371/journal.pone.0292326)
Supplement: Attachment — Submitted filename: Rebuttal letter.docx [file pone.0292326.s005.docx]

PONE-D-23-06866

Protocol for a Randomized Clinical Trial Comparing the Efficacy of Structured Diet (SD) and Regular Therapy (RT) for Children and Adolescents with Malnutrition having Autism Spectrum Disorder (ASD)

PLOS ONE

**Rebuttal letter to the Academic editor and reviewers**

Dear Academic editor

We thank you for your contribution to improving the quality of the paper. In response to your invitation to submit a revised version of the manuscript, we have revised the manuscript addressing every comment.

Journal requirements:

When submitting your revision, we need you to address these additional requirements.

1. Please ensure that your manuscript meets PLOS ONE's style requirements, including those for file naming. The PLOS ONE style templates can be found at

https://journals.plos.org/plosone/s/file?id=wjVg/PLOSOne_formatting_sample_main_body.pdf and

<https://journals.plos.org/plosone/s/file?id=ba62/PLOSOne_formatting_sample_title_authors_affiliations.pdf>

Response: We have addressed the files naming

2. **We note that the grant information you provided in the ‘Funding Information’ and ‘Financial Disclosure’ sections do not match.**

Response: We have revised and written details during the re-submission.

3. PLOS requires an **ORCID iD for** the corresponding author in Editorial Manager on papers submitted after December 6th, 2016. Please ensure that you have an ORCID iD and that it is validated in Editorial Manager. To do this, go to ‘Update my Information’ (in the upper left-hand corner of the main menu), and click on the Fetch/Validate link next to the ORCID field. This will take you to the ORCID site and allow you to create a new iD or authenticate a pre-existing iD in Editorial Manager. Please see the following video for instructions on linking an ORCID iD to your Editorial Manager account: <https://www.youtube.com/watch?v=_xcclfuvtxQ>

Response: Added ORCID IDs

Additional Editor Comments:

1. Can you clearly stated please, how to evaluate or determine the socio-demographics related to malnutrition for ASD?

Response: First objective of the study was revised to determine the socio-demographics and anthropometric measures related to malnutrition for ASD in Bangladesh, The intended variables can be birth history, mother's health status during pregnancy (history of infection, trauma), living environment (urban or rural), family status (joint or single), parental status (separated or conjugal), caregiver (parent, family member or caregiver), living environment, attending school, attending rehab or dietary sessions, sports and amusement facility, age, gender, Body Mass Index. The previous studies found a relationship between these factors <https://www.ncbi.nlm.nih.gov/pmc/articles/PMC4496734/>

2. Can you defined the specific ASD types and their criteria which will be involved in the study?

Response: Details have been described in the diagnosis section. In the DSM-5, four previous types of Autism in DSM-IV (Autistic disorder, Asperger syndrome, childhood disintegrative disorder, and pervasive developmental disorder- not otherwise specified (PDD-NOS) were merged in an umbrella term Autism spectrum disorder (ASD). Also, the first diagnostic category of ASD indicates deficits in social communication and interaction. For an ASD diagnosis, all of the following symptoms must be present (1) impairments in socio-emotional engagement with another person, (2) problems in nonverbal behaviors during communication, (3) problems in making or continuing communication with others. The presence of two or more of the following symptoms associated with limited interest and repetitive behaviors is required for the second domain as (1) persistent or repetitive speech, movement, or holding an object, (2) adherence to routines, rituals of verbal or nonverbal behavior patterns, or excessive resistance to change, (3) abnormal fixated interests or intensity of focus, (4) more or less reactivity to sensory input, or (5) unusual interest in sensory aspects of the environment. According to the third category, symptoms must appear in infancy but may not completely develop until societal demands exceed capacity. We will not focus on any type according to DSM-IV, we will focus on the Autism spectrum according to DSM-V.

3. Clearly state the reason why shall you select the sample only from one single center?

Response: Explained in the revised manuscript in the study setting section as “Two study centers of an organization (one for control and one for experimental) were selected because of the availability of a specialist multi-disciplinary team of physiotherapist, occupational therapist, speech and language therapist, child psychologist, educational psychologist, nutritionist, and special educators were available all together in that specialized organization.”

4. Did you calculated the sample size for this RCT? If yes then clearly state this.

Response: Revised and clarified the sample size calculation as “Sample size calculation has been performed through the software ClinCalc [S1 File] estimating the key outcome as the score of the Gilliam Autism Rating Scale (GARS 2) second edition. 24,31 Sample size has been calculated as the anticipated minimal clinically important differences (MCID) of GARS-2 24 were estimated as 4.7±1.7 (0-10 Hiva scale converted from 0-126 GARS 2 score) with a baseline of 25% minimal clinical improvement, enrolment ratio 1:1, 80% power, and with the alpha value 0.05, the total sample stands as of 66. For safety, we will enroll 70 ASD adolescents with a minimum number of 35 participants in each group.”

5. You mentioned that you have received partial grant but why you did not acknowledge this?

Response: We have revised the funding information section as “Partial funding of the trial was funded by the Bela Health and Education Foundation (BHEF), Grant number BHEF/2022/9/G2. The fund was specified for the identification and intervention provision for Adolescents with Autism Spectrum Disorder in Bangladesh. Publication or any other costs are self-funded by the authors.

6. You mention that you are doing study for Children and Adolescents with Malnutrition having Autism Spectrum Disorder (ASD) but why you selected 13 to 18 years aged participants.

Response: Initially, we screened children and adolescents, but to keep the intervention protocol uniform for the participants in the means of rehabilitation and dietary interventions, we specified the ages 13-18 years. With your comments, we have revised the title and the manuscript as “Protocol for a Randomized Clinical Trial Comparing the Efficacy of Structured Diet (SD) and Regular Therapy (RT) for Adolescents with Malnutrition having Autism Spectrum Disorder (ASD)”. According to WHO, adolescent's age range is 10-19 years <https://www.who.int/health-topics/adolescent-health#tab=tab_1>

Response to Reviewer

Dear Reviewer,

We appreciate your time and scholarly comments to improve the quality of the manuscript. We have revised the manuscript and addressed all of your comments.

**Comments to the Author**

1. Does the manuscript provide a valid rationale for the proposed study, with clearly identified and justified research questions?

The research question outlined is expected to address a valid academic problem or topic and contribute to the base of knowledge in the field.

Reviewer #1: Yes

2. Is the protocol technically sound and planned in a manner that will lead to a meaningful outcome and allow testing the stated hypotheses?

The manuscript should describe the methods in sufficient detail to prevent undisclosed flexibility in the experimental procedure or analysis pipeline, including sufficient outcome-neutral conditions (e.g. necessary controls, absence of floor or ceiling effects) to test the proposed hypotheses and a statistical power analysis where applicable. As there may be aspects of the methodology and analysis which can only be refined once the work is undertaken, authors should outline potential assumptions and explicitly describe what aspects of the proposed analyses, if any, are exploratory.

Reviewer #1: Partly

Response: We have revised to improve the manuscript

3. Is the methodology feasible and described in sufficient detail to allow the work to be replicable?


Descriptions of methods and materials in the protocol should be reported in sufficient detail for another researcher to reproduce all experiments and analyses. The protocol should describe the appropriate controls, sample size calculations, and replication needed to ensure that the data are robust and reproducible.

Reviewer #1: Yes

4. Have the authors described where all data underlying the findings will be made available when the study is complete?

The [PLOS Data policy](https://journals.plos.org/plosone/s/materials-and-software-sharing) requires authors to make all data underlying the findings described in their manuscript fully available without restriction, with rare exception, at the time of publication. The data should be provided as part of the manuscript or its supporting information, or deposited to a public repository. For example, in addition to summary statistics, the data points behind means, medians and variance measures should be available. If there are restrictions on publicly sharing data—e.g. participant privacy or use of data from a third party—those must be specified.

Reviewer #1: Yes

5. Is the manuscript presented in an intelligible fashion and written in standard English?

PLOS ONE does not copyedit accepted manuscripts, so the language in submitted articles must be clear, correct, and unambiguous. Any typographical or grammatical errors should be corrected at revision, so please note any specific errors here.

Reviewer #1: Yes

6. Review Comments to the Author

Please use the space provided to explain your answers to the questions above and, if applicable, provide comments about issues authors must address before this protocol can be accepted for publication. You may also include additional comments for the author, including concerns about research or publication ethics.

You may also provide optional suggestions and comments to authors that they might find helpful in planning their study.


(Please upload your review as an attachment if it exceeds 20,000 characters)

Reviewer #1: This is a study protocol to conduct a randomized controlled clinical trial to compare the effectiveness of combined RT + SD therapy, compared to UD, for children/adolescents with ASD. The study was registered within the Indian clinical trial registry, and was also approved by the respective Ethics board. The study objectives are on target, and the study design looks adequate. ASD is an important disease where more research is needed; hence, the study objectives are on target. However, I have some concerns/comments in the statistical design, analytical framework, etc, which may require closer attention:

1. Randomization: More details on the randomization procedure is needed; in particular, this reviewer thought that a blocked randomization would be helpful to achieve a 1:1 balanced recruitment within the 2 groups.

Response: We have revised the section “Recruitment and randomization”. Also added the line “In the group allocation process, block randomization will be employed to ensure the balanced recruitment for two groups.”

2. Outcome variables: Clearly state what will be measured, i.e., the outcome variables, both primary, and if necessary, the secondary. It was not clear, given that a bunch of variables to be collected has been mentioned.

Response: We have made two sections as explanatory variables (socio-demographic information and diagnosis of ASD) and primary outcome variables (GARS-2 and anthropometric measures)

3. Sample size/Power: The sample size/power writeup is half-hearted. In any randomized trial, it is recommended to power the study based on the primary outcome variable, using 5% significance, using a desired (moderate) effect size, and a specific statistical test, such as a 2-sample t-test, or a relevant nonparametric 2-sample test (if one suspects non-Gaussianity of the primary outcome/response variable). None of that has been specified; only what I see is 35 in each group (total of 70!).

Response: Revised and clarified the sample size calculation as “Sample size calculation has been performed through the software ClinCalc [S1 File] estimating the key outcome as the score of the Gilliam Autism Rating Scale (GARS 2) second edition. 24,31 Sample size has been calculated as the anticipated minimal clinically important differences (MCID) of GARS-2 24 were estimated as 4.7±1.7 (0-10 Hiva scale converted from 0-126 GARS 2 score) with a baseline of 25% minimal clinical improvement, enrolment ratio 1:1, 80% power, and with the alpha value 0.05, the total sample stands as of 66. For safety, we will enroll 70 ASD adolescents with a minimum number of 35 participants in each group.”

4. Statistical Analysis: Looks adequate; any reasoning, why a formal mixed-effects modeling will not be conducted (controlling for possible covariates), compared to a repeated-measures ANOVA.
Also, Bonferroni adjustments are often harsh; I would recommend going with false discovery rates (FDRs).

Response: We assume no missing data in the post-test and follow-up, expect a normal distribution through block randomization, a simple post-hoc analysis (pre-post, post-follow-up), time as a category, and only two repetitions of data collection. All these factors suggest in favor of repeated-measures ANOVA. <https://www.theanalysisfactor.com/six-differences-between-repeated-measures-anova-and-linear-mixed-models/>

However, formal mixed-effects modeling, and false discovery rates both are great options. We thank the reviewers for the comment “Looks adequate” and for the suggestions, we can revise it until it is mandatory.

5. Writing style:


The Discussion section should clearly state that the findings of this study would be only limited to the enrolled sample (which has a moderate sample size) of Bangladeshi subjects. The authors should allude to future trials with larger sample sizes (under medium to large effect sizes), and covering other populations, to establish the credibility of their findings.

Response: Revised in the discussion section between lines 365 and 368.
